# Genome-Wide Identification of Molecular Mimicry Candidates in Parasites

**DOI:** 10.1371/journal.pone.0017546

**Published:** 2011-03-08

**Authors:** Philipp Ludin, Daniel Nilsson, Pascal Mäser

**Affiliations:** 1 Institute of Cell Biology, University of Bern, Bern, Switzerland; 2 Swiss Tropical and Public Health Institute, Basel, Switzerland; 3 University of Basel, Basel, Switzerland; The University of Maryland, United States of America

## Abstract

Among the many strategies employed by parasites for immune evasion and host manipulation, one of the most fascinating is molecular mimicry. With genome sequences available for host and parasite, mimicry of linear amino acid epitopes can be investigated by comparative genomics. Here we developed an *in silico* pipeline for genome-wide identification of molecular mimicry candidate proteins or epitopes. The predicted proteome of a given parasite was broken down into overlapping fragments, each of which was screened for close hits in the human proteome. Control searches were carried out against unrelated, free-living eukaryotes to eliminate the generally conserved proteins, and with randomized versions of the parasite proteins to get an estimate of statistical significance. This simple but computation-intensive approach yielded interesting candidates from human-pathogenic parasites. From *Plasmodium falciparum*, it returned a 14 amino acid motif in several of the PfEMP1 variants identical to part of the heparin-binding domain in the immunosuppressive serum protein vitronectin. And in *Brugia malayi*, fragments were detected that matched to periphilin-1, a protein of cell-cell junctions involved in barrier formation. All the results are publicly available by means of mimicDB, a searchable online database for molecular mimicry candidates from pathogens. To our knowledge, this is the first genome-wide survey for molecular mimicry proteins in parasites. The strategy can be adopted to any pair of host and pathogen, once appropriate negative control organisms are chosen. MimicDB provides a host of new starting points to gain insights into the molecular nature of host-pathogen interactions.

## Introduction

Endoparasites are confronted with host defenses at multiple levels: physical barriers, innate immunity, and adaptive immune responses need to be overcome in order to successfully establish an infection and proliferate inside a host. Antigenic variation to escape humoral responses is well documented for the malaria parasites, *Giardia*, African trypanosomes, etc. Further strategies for immune evasion or immune suppression are less well understood. Molecular mimicry as a strategy for immune evasion and host manipulation is well known from viruses [1], [2]. While many viruses have a natural propensity to acquire genetic material or proteins from the host cell upon formation of virions, others have by themselves evolved surface proteins for mimicry, e.g. the chemokine receptors of cytomegalovirus [3]. The term molecular mimicry was coined by R. Damian in 1964 and defined as the sharing of antigens between parasite and host [4]. We refer here to molecular mimicry as the display of any structure by the parasite that (i) resembles structures of the host at the molecular level and (ii) confers a benefit to the parasite because of this resemblance. The potential benefits of molecular mimicry include camouflage – as exemplified by the concept of ‘eclipsed antigens’ which are not recognized as such by the host's immune system due to their similarity to host antigens [5] – and cytoadherence. For intracellular parasites, cytoadherence is a prerequisite to infection. Trypomastigote *T. cruzi* adhere to fibroblasts via the fibronectin receptor, and exogenous peptides with fibronectin RGD motifs inhibited host cell invasion [6], [7]. Cytoadherence of *P. falciparum*-infected erythrocytes to microvascular endothelium contributes to cerebral malaria pathology. *P. falciparum* erythrocyte membrane protein 1 (PfEMP1, encoded by the *var* genes) interacts with adhesion molecules such as ICAM-1, CD36, or thrombospondin via different domains [8], [9]. Endothelial adherence prevents the infected erythrocytes from passage to the spleen where they would be eliminated. A third reason why parasites might mimic host molecules is signaling. Parasites may mimic hormone receptors to respond to signals from the host, or mimic hormones to send signals to the host. Functional homologues of the mammalian epidermal growth factor (EGF) receptor were described from trypanosomes [10], [11] and helminths [12], [13]. *Plasmodium* spp. possess at least two surface proteins with EGF motifs, one (Pfs25) expressed in the mosquito [14], the other (MSP1) in the blood-stages where it is critical for erythrocyte invasion [15], [16]. Schistosomes send immunosuppressory signals in the form of neuropeptides to both the definite host (man) and the intermediate host (snail) [17]. There are extreme cases of behavioral manipulation of the host by the parasite such as the suicidal diving of grasshoppers infected by hairworms, and there too molecular mimicry is likely to play a role [18].

The first evidence for molecular mimicry between parasite and host came from immunological studies on antisera that cross-reacted with parasite and host. *Ascaris lumbricoides* was found to possess A- and B-like blood group antigens [19]. This was confirmed by more recent studies, which suggested that these antigens had been acquired from host blood [20]. Biosynthesis of human blood group-like antigens was described for *Schistosoma mansoni* [21], [22] and *Fasciola hepatica* [23]. However, the function of these antigens produced by the parasite remains to be elucidated. More recently, tools other than antisera were used to address molecular mimicry between parasite and host. Molecular cloning of the involved genes [24], [25], elucidation of polysaccharide structures [26], use of monoclonal antibodies [27], [28] and synthetic peptides [29] have all contributed to a wealth of evidence that endoparasites take advantage of molecular mimicry to survive in their hosts (see also Table 1). Recurring targets for mimicry by bloodborne pathogens are the components of the complement system, growth hormones and their receptors, and cell adhesion molecules [30]. A parasite's ability to perform molecular mimicry may stem from either having acquired macromolecules from the host (transfer) or from adaptive evolution of the mimicking structures (convergence). Both scenarios are supported by multiple examples from parasites (Table 1). With the rapidly growing number of fully sequenced genomes, direct comparison between host and parasite protein sequences provides a powerful tool to identify molecular mimicry candidates. To our knowledge, however, there has been no systematic approach to study molecular mimicry since parasitology entered the post-genomic era.

**Table 1:** Possible mechanism for molecular mimicry and examples from pathogens.

Macromolecule	Mimicry by transfer	Mimicry by convergence
**Nucleic acid**	*Schistosoma mansoni* possesses a CRIT gene which shares 98% identical nucleotides with the human orthologue [25].	The 3′UTR of the RNA genome of barley yellow dwarf virus mimics the m^7^G cap of eukaryotic mRNA to stimulate translation [59].
**Protein**	Pathogenic bacteria, *E. granulosus* and *O. volvulus* decorate themselves with inhibitors of the complement cascade sequestered from the blood [43], [60], [61], [62].	A 18 aa motif in *P. falciparum* CSP is nearly identical to the cytoadhesive region of mammalian thrombospondin [49] and was shown to bind to hepatocytes [63].
**Sugar**	Trans-sialidases transfer sialic acid from host cells to the surface of the parasite. *T. cruzi* trans-sialidase is a virulence factor in mammals [64]; *T. brucei* trans-sialidase is required for survival in the tsetse fly [65].	Several pathogenic helminths synthesize the Forssman antigen (globopentosylceramide) [21], [66], a glycolipid implicated in cell adhesion and the formation of tight junctions [67].

Mimicry by transfer of nucleic acids or convergence of proteins can be identified *in silico* by comparative genomics (CRIT: Complement C2 receptor inhibitor trispanning (CRIT), C4BP: m^7^G, 7-methyl guanosine; CSP, circumsporozoite protein; Complement-binding protein, CR1: Complement receptor 1, FHL-1: factor-H-like protein-1, fH: factor H, MCP: Membrane cofactor protein, DAF: Decay-accelerating factor).

Here we develop an *in silico* pipeline to identify molecular mimicry candidates from parasites. In brief, proteome-wide blast surveys were performed with either whole proteins or with overlapping protein fragments to identify similar epitopes in parasite and host. This approach warrants that all linear amino acid epitopes which share significant similarity between parasite and host will be discovered. Searches against control proteomes of free-living eukaryotes served as negative controls to exclude proteins that are generally conserved across phyla, while searches with random sequences allowed to estimate statistical significance. The results are made available by means of an online database for molecular mimicry candidate proteins in pathogens.

## Results and Discussion

### Molecular mimicry surveys with full length protein sequences

**Figure 1:**
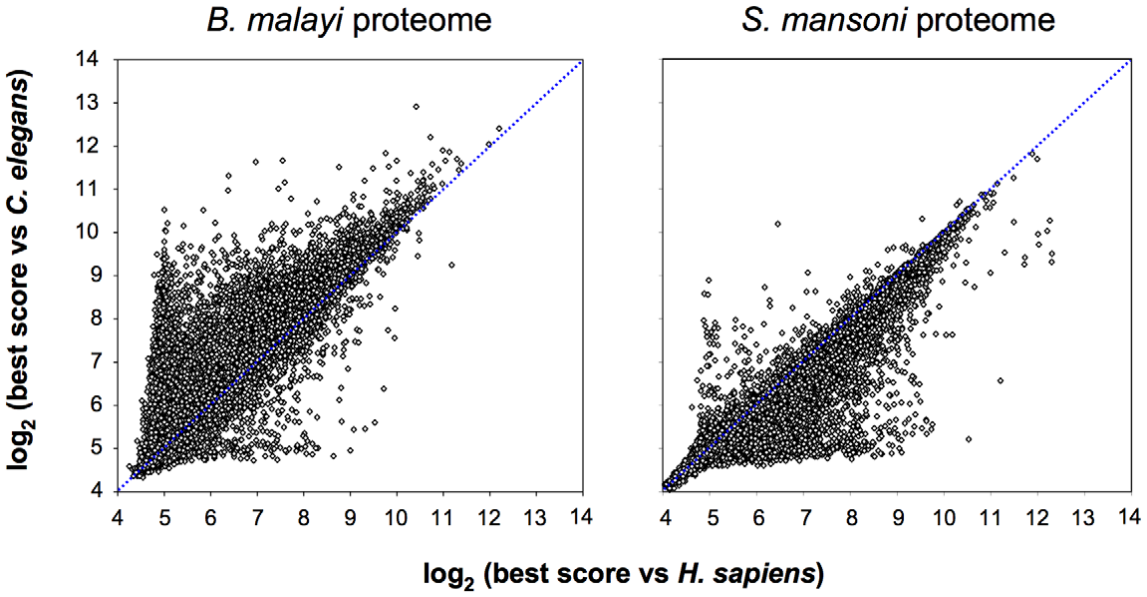
Scatter plot of the blast scores of all proteins from *B. malayi* (left) and *S. mansoni* (right) vs. the host *H. sapiens* (x-axis) and the control *C. elegans* (y-axis). Points below the blue dotted line represent parasite proteins with better scores to *H. sapiens* than to *C. elegans*.

In pilot surveys for molecular mimicry candidates we concentrated on endoparasitic helminths since (i) they are known masters of immune evasion and host manipulation, and (ii) a convenient negative control is available in the form of the free-living nematode *C. elegans*. In principal, a mimicry candidate is a parasite protein or motif which bears a high degree of resemblance to a protein of the host but not to those of unrelated control species. Such proteins are readily identified by proteome-wide blast surveys. In a first trial, we ran every predicted protein of *Brugia malayi* with blastp against the proteomes of *H. sapiens* and *C. elegans*. As expected, the *B. malayi* proteins returned significantly (p<0.0001, two-tailed Wilcoxon test) higher scores against *C. elegans* than against *H. sapiens*. There were only few *B. malayi* proteins which scored better against the human host (Figure 1, left). The converse picture emerged when the same procedure was carried out with *Schistosoma mansoni* (Figure 1, right) or *S. japonicum* (not shown), where the parasite proteins generally were more similar to human than to *C. elegans* proteins (p<0.0001, two-tailed Wilcoxon test). The systemic nature of the phenomenon (Figure 1, right) speaks against molecular mimicry as the underlying selective force since it involves too many housekeeping proteins that do not interact with the host. *C. elegans* and *S. mansoni* are from different metazoan clades, the ecdysozoa and the lophotrochozoa, respectively [31]. While the *S. mansoni* proteins were also more similar to *D. melanogaster* than to *C. elegans* proteins, the overall similarity to human proteins was still the most pronounced (not shown).

**Figure 2:**
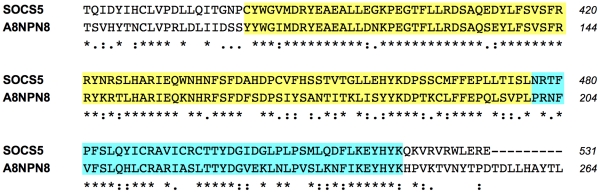
ClustalW alignment of the candidate mimicry region in A8NPN8 from *B. malayi* to *H. sapiens* SOCS5. The SH2 domain is shaded in yellow, the SOCS box domain in blue. The N-terminal parts of the two proteins do not share any similarity (not shown).

The two-dimensional blastp approach allowed to graphically divide the proteome of *B. malayi* into separate quadrants: parasite-specific proteins (lower left in Figure 1, left), generally conserved proteins such as tubulin or ubiquitin (upper right), nematode-specific proteins (upper left), and mimicry candidates (lower right). However, this rough subdivision is prone to false positives caused by the well documented phenomenon of gene loss in *C. elegans* [32]. In order to eliminate proteins which are generally conserved, the negative control was refined to include – in addition to *C. elegans* – a panel of unrelated, free-living eukaryotes whose genomes have been sequenced: *Saccharomyces pombe*, *Arabidopsis thaliana*, *Ciona intestinalis*, and *Trichoplax adhaerens* (Table 2). For the detection of mimicry candidates we focused on human-pathogenic endoparasites known for their mastery in immune evasion, namely *Brugia malayi*, *Schistosoma mansoni*, *Plasmodium falciparum*, *Leishmania major*, *Cryptosporidium parvum*, *Trichomonas vaginalis* and *Trypanosoma cruzi* (Table 2). The predicted proteomes of the parasites were run as blast queries against the control proteomes and against *H. sapiens*. Molecular mimicry candidates were defined as parasite proteins with (i) a blastp score above 100 to the best hit in the human proteome and (ii) a score in *H. sapiens* at least two-fold higher than the best score achieved in the control proteomes. This search returned 84 hits, most of which from *S. mansoni* (52) and *B. malayi* (15; Table S1). One hit from *B. malayi* was a predicted protein (A8NPN8) with strong similarity to human suppressor of cytokine signaling 5 (SOCS5), in particular to the SH2 domain and the SOCS box (Figure 2). Human SOCS5 was shown to inhibit the IL-4 pathway in T helper cells, promoting T_H_1 differentiation [33]. The SH2 domain recognizes the target molecule and the SOCS box recruits the ubiquitin complex that mediates proteosomal degradation of the target [34]. SOCS proteins being crucial regulators of both innate and adaptive immunity, the SOCS5-like protein from *B. malayi* is an interesting candidate. However, it does not carry an export signal and it is therefore not clear how it should interact with host proteins. Possibly, it is released when parasites die.

**Table 2:** Organisms used in this study.

Species	Proteins	Source	Ref.
*Brugia malayi*	11551	Uniprot	[68]
*Cryptosporidium parvum*	3805	CryptoDB	[69]
*Giardia lamblia*	5901	GiardiaDB	[70]
*Leishmania major*	8406	TritrypDB	[71]
*Plasmodium falciparum*	5479	PlasmoDB	[72]
*Schistosoma mansoni*	13157	Sanger	[73]
*Trichomonas vaginalis*	50155	Uniprot	[74]
*Trypanosoma cruzi*	23031	TritrypDB	[75]
*Homo sapiens*	20298	Uniprot	[76]
*Aedes aegypti*	16531	Vectorbase	[77]
*Anopheles gambiae*	14103	Vectorbase	[78]
*Arabidopsis thaliana*	36671	EBI	[79]
*Caenorhabditis elegans*	24143	Wormbase	[80]
*Ciona intestinalis*	15852	JGI	[81]
*Schizosaccharomyces pombe*	4977	EBI	[82]
*Trichoplax adhaerens*	11585	Uniprot	[83]

Parasite (top), host (middle), and negative control species (bottom), their predicted number of protein-coding genes, and source of the predicted proteome file (EBI: European Bioinformatics Institute, JGI: Joint Genome Institute).

The known mimicry candidate CRIT (complement C2 receptor inhibitory trispanning, Table 1), which is almost identical between *S. mansoni* and *H. sapiens* [35], was not identified here because human CRIT is not included in the reviewed human proteome from Swissprot (Table 2). Searching against the whole human Uniprot dataset readily returned *S. mansoni* CRIT as the top hit. In the classical complement pathway CRIT blocks the formation of C3 convertase by decreasing the association of C2 with C4b; once C2 is attached to the receptor, it cannot be cleaved by C1 to produce C2a and C2b and thus C3 convertase is no longer formed – the classical pathway is disrupted [25]. It is easy to conceive that a parasite gains an advantage in the human body by exhibiting CRIT and diminishing the proinflammatory response. Based on the high level of DNA similarity *S. mansoni* is thought to have acquired the CRIT gene by horizontal transfer [25], [35]. However, while CRIT orthologues are present in all of the sequenced *Schistosoma* species and in *T. cruzi*, the only mammals which possess CRIT are man and rat (Figure S1). This enigmatic distribution can only be explained by multiple instances of gene transfer or gene loss in mammals. Postulating a minimal number of horizontal transfers, a parsimonial interpretation would place the origin of the CRIT gene to schistosomes. The gene could have been acquired (exapted) from the parasites by *H. sapiens* and *R. norvegicus* independently, and finally picked up by *T. cruzi* from a mammalian host. In this scenario, only the CRIT of *T. cruzi* would be a case of molecular mimicry.

### Molecular mimicry surveys with fragmented protein sequences

**Figure 3:**
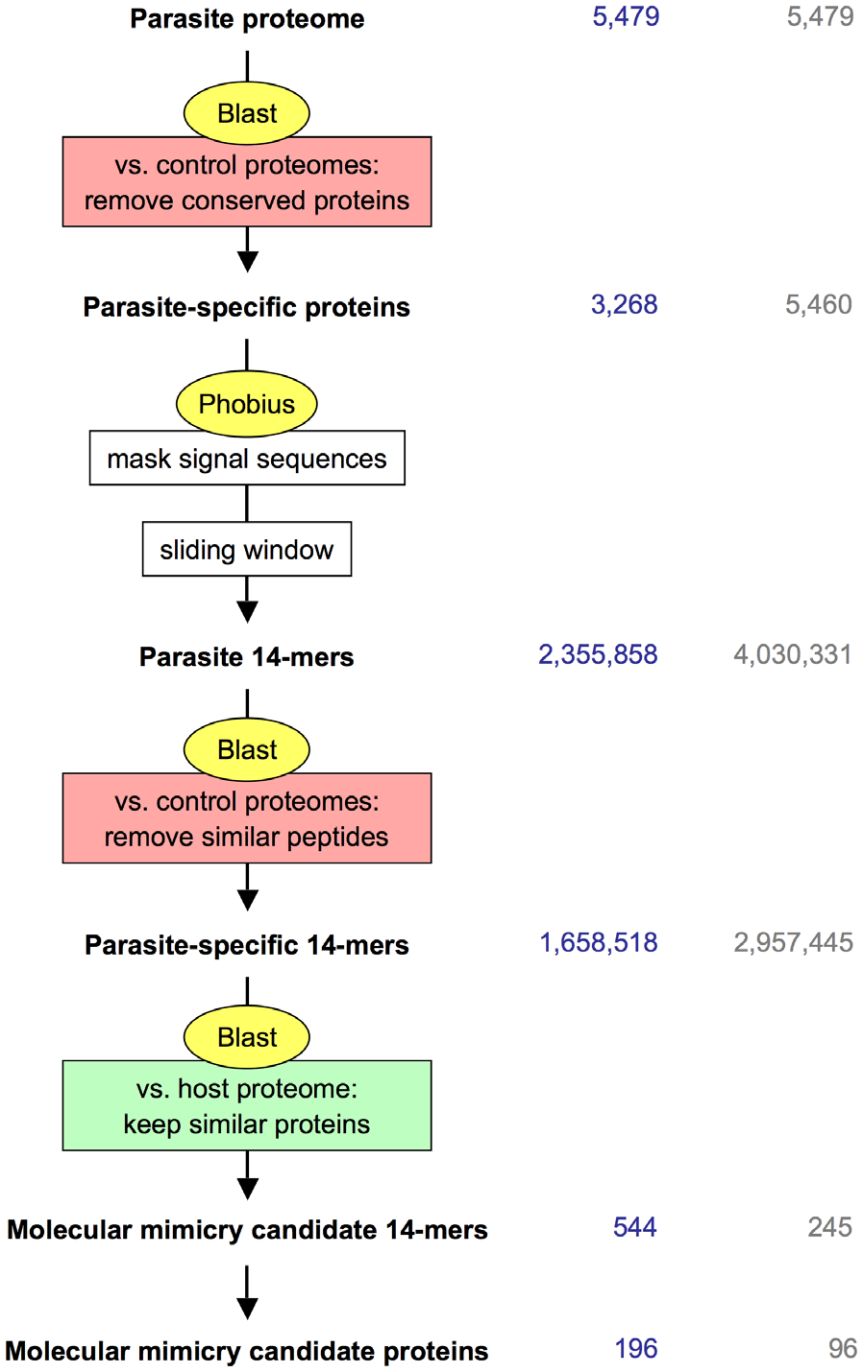
The *in silico* pipeline for identification of molecular mimicry candidates from parasites. See Methods for details. The process is illustrated with the actual numbers from the analysis of the *P. falciparum* proteome in blue, respectively a randomized version of it in grey, vs. the host *H. sapiens*.

**Figure 4:**
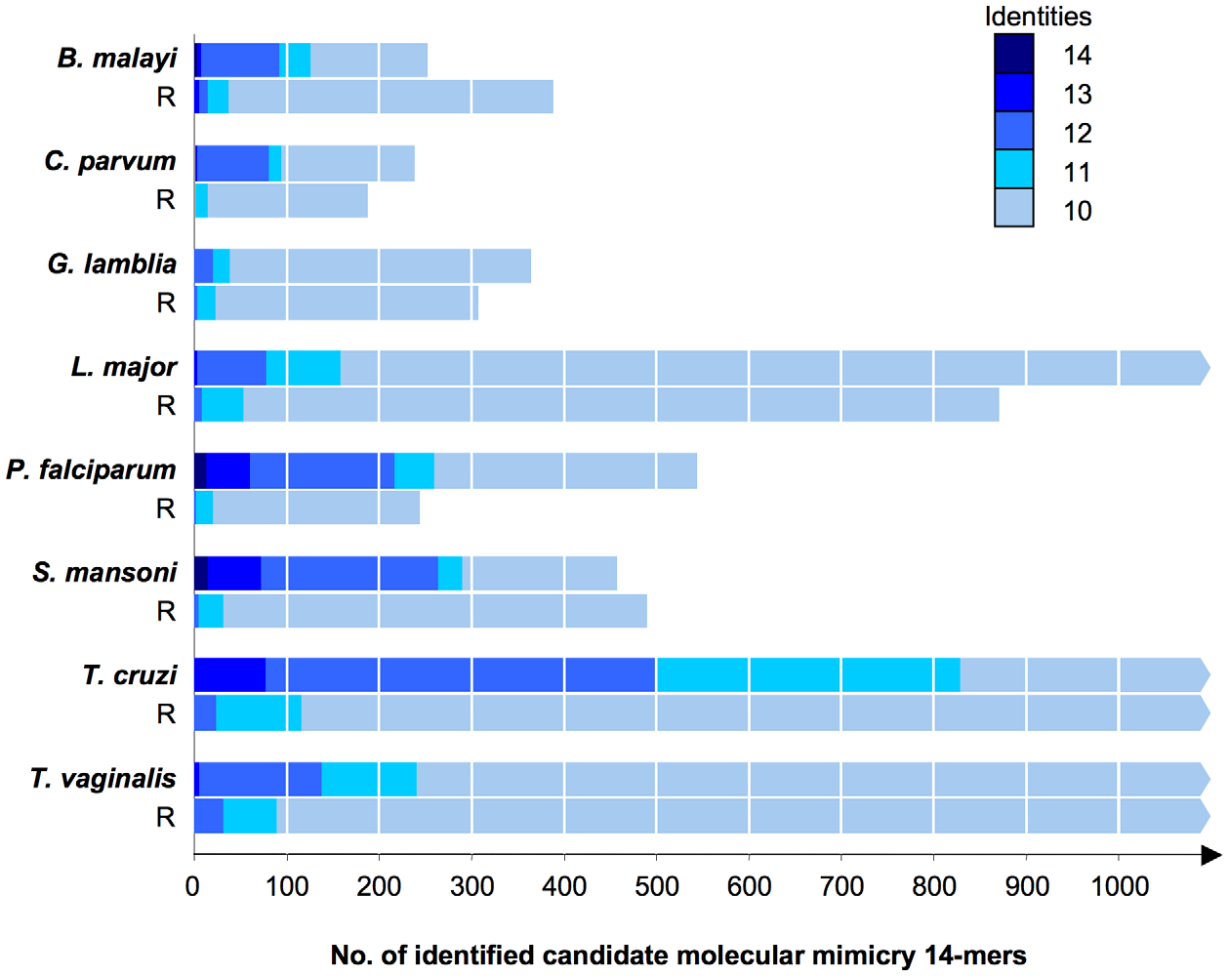
Numbers of identified candidate molecular mimicry 14-mers from parasite proteomes and randomized versions thereof (R). Numbers of amino acid identities between the 14-mers and their best hit in the human proteome are color-coded as indicated.

Several known cases of molecular mimicry from parasites (Table 1) involve shorter peptides, e.g. the thrombospondin motif in *P. falciparum* circumsporozoite protein CSP. Such mimicry candidates would not be detected with the above approach using full-length protein sequences. Thus we refined the systematic survey and developed a peptide-based pipeline for detection of mimicry candidates as outlined in Figure 3. In brief, the parasite proteins were converted to a series of overlapping 14-mers, each of which was searched with ungapped blastp against the control proteomes *C. elegans, S. pombe*, *A. thaliana*, *C. intestinalis*, or *T. adhaerens*. The 14-mers with high similarity to any sequence of the controls were filtered out using an empirically developed scheme (Figure S2). The remainder of the 14-mers was screened, again with ungapped blastp, against the *H. sapiens* proteome and those exhibiting strong similarity (Figure S2) to a human sequence were identified as molecular mimicry candidates. For this approach, predicted N-terminal protein export signal sequences were removed since they resemble each other and might produce false positive hits. Parasite 14-mers with 100% identity to a human protein were obtained from *B. malayi* (4), *C. parvum* (1), *P. falciparum* (13) and *S. mansoni* (15). 14-mers with 13 identical residues to a human protein were found in all parasites except *G. lamblia*. The number of hits is summarized in Figure 4. As a control, the same approach (Figure 3) was carried out with versions of the pathogen proteomes where every sequence had been scrambled randomly. This yielded not a single 14-mer of 100% identity to a human protein over all the parasites tested, and only 4 with 13 identities in, underscoring the statistical significance of the identified mimicry candidates. The largest differences between real and randomized proteins were observed for the helminths *B. malayi* and *S. mansoni*, and for *P. falciparum*. Selected mimicry candidates from these parasites are listed in Table 3. The selection was based on number of identical residues, Shannon-entropy of the respective 14-mer as a measure of sequence heterogeneity, and GO terms associated with the hit in the human proteome. An overview of all the high-level GO terms of the human proteins which were matched with mimicry candidates from parasites is shown in Table S2. The mimicry candidates of *P. falciparum* enriched for ‘Cellular component biogenesis’, ‘Localization’, and ‘Growth’, while for the helminths *B. malayi* and *S. mansoni* ‘Biological adhesion’ and ‘Rhythmic process’ were overrepresented in the human hits (compared to the complete human proteome; Table S2).

**Table 3:** Selected mimicry candidates.

Parasite protein	14-mer motif	Ent.	Id.	Human protein
*Bma* A8PSR3, uncharacterized protein	TFGFVTKMLIEKDP	3.24	12	Q8NEY8, periphilin-1
*Bma* A8Q9C9, uncharacterized protein	RKSSQKIRMRDVVL	3.04	12	P00747, plasminogen
*Bma* A8QH34, leucyl aminopeptidase	HLDSWDVGQGAMDD	3.09	14	Q9Y646, plasma glutamate carboxypeptidase
*Bma* A8PP49, pregnancy-associated plasma protein E	CYIYEGDGECEPFE	2.81	12	Q9BXP8, pappalysin-2
*Sma* Smp_111120, insulin receptor kinase substrate	SLEKSQAELKKIRR	2.90	14	Q9UHR4, insulin receptor tyrosine kinase substrate
*Sma* Smp_109770, integrin alpha-4	APNVSMEIMVPNSF	3.09	13	P13612, integrin alpha-4
*Pfa* PF07_0048, PfEMP1	NPEQTPVLKPEEEA	2.90	14	P04004, vitronectin
*Pfa* MAL13P1.34, RED-like protein	SKFMGGDEEHTHLV	3.38	12	Q13123, IK cytokine
*Pfa* PF13_0201, TRAP	WDEWSPCSVTCGKG	3.24	12	Q9HCB6, spondin-1

Hits from *B. malayi* (*Bma*), *S. mansoni* (*Sma*) and *P. falciparum* (*Pfa*) and their human match (Ent, Shannon entropy in bits; Id, number of identities).

Among the most interesting of the identified mimicry candidates was a match of 17 identical amino acids from *B. malayi* to human plasma glutamate carboxypeptidase. The *B. malayi* protein (A8QH34) had been previously detected in excretory-secretory products in abundance [36], [37]. Moreover, the identified candidate has 67% identity to ES-62 from the rodent filarial nematode *Acanthocheilonema viteae* (Uniprot ID O76552), a protein with immunomodulatory impact on different host cells depending on the occurrence of phosphorylcholine [38]. The identified candidate stretch shares 14 identical amino acids with ES-62 of *A. viteae*. Other interesting fragments from *B. malayi* matched to human periphilin-1 (Q8NEY8), a protein of cell-cell junctions in differentiated keratinocytes which was proposed to be involved in barrier formation and epidermal integrity [39], and to plasminogen (P00747), the proenzyme of plasmin which dissolves blood clots and acts as a proteolytic factor in various other processes (Table 3).

**Figure 5:**
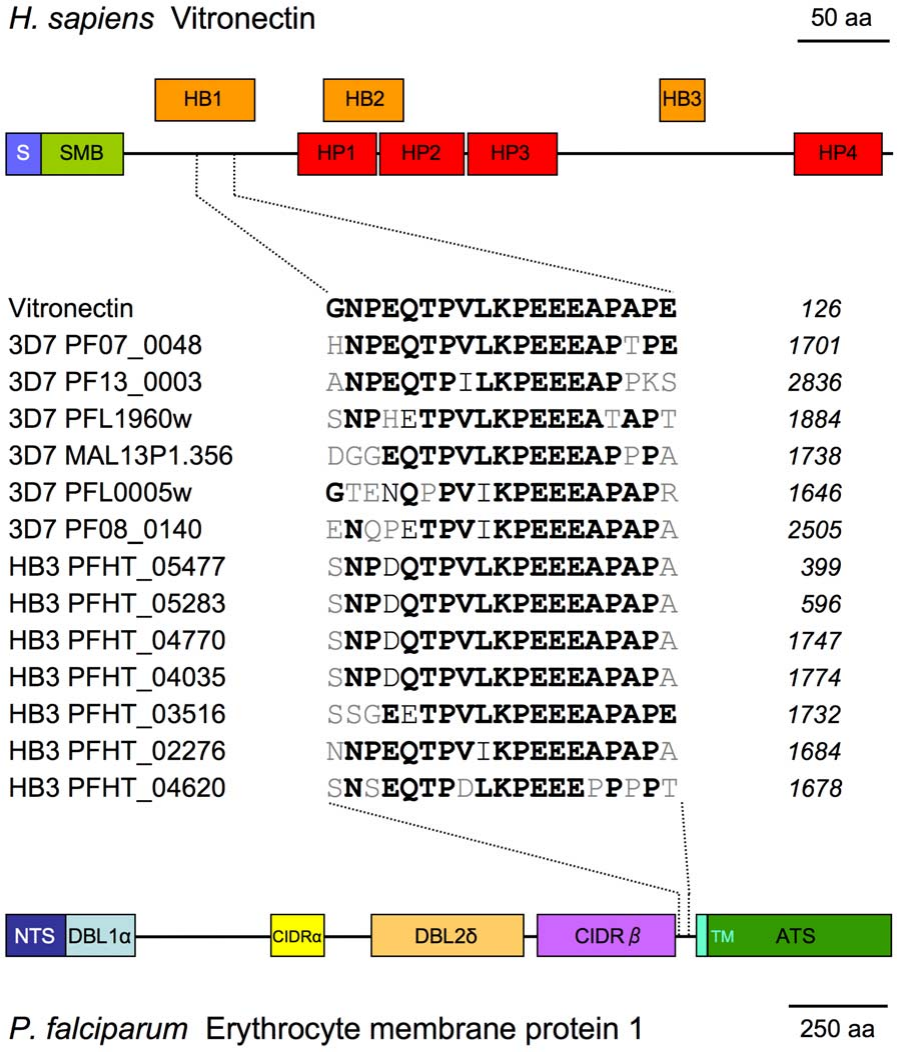
Alignment of human vitronectin (top) and *P. falciparum* PfEMP1 variants (bottom). Identities to vitronectin are printed in bold black, similarities in black. The known vitronectin domains are the signal sequence (blue), somatomedin-B (green), and hemopexin (red). The known PfEMP1 domains are the N-terminal segment (dark blue), Duffy Binding Like α (light blue), cysteine-rich interdomain region α (yellow), Duffy Binding Like 2d (orange), cysteine-rich interdomain region ß (purple), transmembrane domain (cyan), acidic terminal segment (green).

In *P. falciparum*, the peptide-based approach significantly enriched for exoproteins (p<0.0001, two-sided chi square test), i.e. proteins with transmembrane domains or export signal predicted by Phobius [40]. The best hit overall was to human vitronectin. Several of the *var* family gene products turned out to share a stretch of 13 to 16 identical amino acids with vitronectin. The candidate mimicry motif lies in the extracellular part of PfEMP1, close to the predicted transmembrane domain (Figure 5, bottom). The corresponding sequence in vitronectin is in the N-terminal half, in the first of the heparin-binding motifs between the somatomedin and the central hemopexin domains (Figure 5, top). Vitronectin is a multifunctional protein that promotes cell adhesion, stabilizes plasminogen activator inhibitor 1, and inhibits the formation of the pore-forming membrane attack complex (MAC) of the complement system. Vitronectin is abundant in the extracellular matrix and in the serum [41]. Pathogenic bacteria such as *Neisseria meningitides* or *Haemophilus influenzae* decorate themselves with human vitronectin which they acquire form the serum through specific binding partners on their surface [42], [43]. Bacteria also exploit human vitronectin for cytoadhesion and host cell invasion [44]. Malaria-infected erythrocytes, however, tested negative for binding to human vitronectin [45]. We identified six PfEMP1 variants possessing the candidate mimicry motif to vitronectin in the *P. falciparum* strain 3D7 and seven in the strain HB3 (Figure 5). The motif is positionally conserved relative to the transmembrane domain of PfEMP1. Searching the non-redundant protein database of GenBank with the corresponding peptide ‘NPEQTPVLKPEEEAP’ returned significant hits (expectancy <0.001) only from *H. sapiens*, Chimpanzee, Orangutan, and *P. falciparum* (not shown). Interestingly, the genome project of the simian and human malaria parasite *P. knowlesi* had uncovered a candidate molecular mimicry motif to the immunoregulatory host protein CD99 in the extracellular domain of the *kir* gene family products [46].

The fragment-based approach for mimicry candidates in *P. falciparum* also returned a triad between host, vector and parasite. Thrombospondin-related anonymous protein (TRAP, PF13_0201) of *P. falciparum* matched with the human spondin (Q9HCB6) and a hypothetical protein from *A. gambiae* (AGAP012307, not shown). In the human protein, the region lies in the thrombospondin type-I repeat (TSR) domain which binds to heparin sulphate proteoglycans on hepatocytes [47], [48]. This mimicked structure was also found on the circumsporozoite protein (CSP) and has been known for a long time [49]. Whereas CSP mediates the binding of the parasites to the human liver, it is suggested that TRAP is crucial for sporozoite locomotion and cell invasion [50], [51]. Interestingly, the same part of the TSR domain of TRAP has been matched with the *A. gambiae* proteome and it has been demonstrated with loss-of-function mutations that this region is involved in the sporozoite invasion into mosquito salivary glands [52].

### mimicDB - Database for molecular mimicry candidates from pathogens

All mimicry candidates from parasites to mammalian and insect hosts (Table 2) were stored in a relational database, mimicDB, which is publicly accessible via <http://mimicdb.scilifelab.se>. The database was designed for ease of community access to the mimicry data (Figure S3). It can be queried using keywords from gene description, different formats of gene and protein accession numbers and names, and in general on free text on the available data. GO terms are tightly integrated into the database, and queries can be made both on leaf-terms as well as directly onto broader categories higher up in the hierarchy. The queries can be restricted to species using special qualifiers. From the resulting tables, links are provided directly to entries in large public databases (Uniprot, NCBI) as well as to detailed sequence views. Predicted protein motifs and signal peptides are visualized on the source and target sequences together with the candidate mimicry motifs.

### Conclusion

To our knowledge this is the first *in silico* survey for molecular mimicry candidates in parasites. Its systematic, genome-wide nature warrants that all linear amino acid epitopes involved in molecular mimicry between a given parasite and its host are going to be detected. False positive hits can be tracked by including the appropriate controls: proteomes of free-living species to eliminate the proteins which are generally conserved across phyla, and scrambled versions of the parasite proteomes to estimate for random hits resulting from the sheer number of analyzed sequences. False negatives are more problematic; mimicry by non-linear epitopes composed from amino acids of separate folds (or even separate polypeptides) will not be recognized, and neither are glycosylated epitopes (Table 1). Nevertheless, there are examples of molecular mimicry by linear epitopes which are straightforward to detect by comparative genomics as performed here. Proof of concept was obtained from the fact that the known molecular mimicry motif in TRAP (thrombospondin-related anonymous protein) from *P. falciparum* was detected readily. Many new molecular mimicry candidates were discovered from human parasites, in particular from *B. malayi*, *S. mansoni* and *P. falciparum*, most notably a sequence shared between human vitronectin and several of the *P. falciparum* erythrocyte membrane protein 1 variants. All the identified mimicry candidates are stored in a relational database called mimicDB and searchable on-line. We hope that mimicDB will stimulate research into molecular mimicry of parasites. Given its numerous potential benefits – camouflage, cytoadherence, manipulation of host signaling – molecular mimicry may well be much more common among parasitic microorganisms than currently known.

## Materials and Methods

### Proteome files

Predicted proteins from completely sequenced genomes (Table 2) were obtained from ftp.ebi.ac.uk (*Arabidopsis thaliana*, *Schizosaccharomyces pombe*), www.tritrypdb.org (*Leishmania major*, *Trypanosoma cruzi*), www.cryptodb.org (*Cryptosporidium parvum*) www.giardiadb.org (*Giardia lamblia*) www.plasmodb.org (*Plasmodium falciparum* 3D7), www.broadinstitute.org (*Plasmodium falciparum* HB3), ftp.vectorbase.org (*Aedes aegypti*, *Anopheles gambiae*), ftp.wormbase.org (*Caenorhabditis elegans*), ftp.sanger.ac.uk (*Schistosoma mansoni*), ftp.jgi-psf.org (*Ciona intestinalis*), and www.uniprot.org (*Brugia malayi*, *Homo sapiens*, *Trichomonas vaginalis*, *Trichoplax adhaerens*).

### Algorithm

BLAST 2.2.17 [53] was obtained from ftp.ncbi.nlm.nih.gov, Phobius 1.01 [40] from <phobius.sbc.su.se>. Automated detection of molecular mimicry candidates as depicted in Figure 3 was performed with Perl scripts, available on request. First, those of the predicted parasite proteins which are generally conserved among eukaryotes were sorted out based on full-length blastp searches against the proteomes of *C. elegans*, *C. intestinalis*, *T. adhaerens*, *S. pombe and A. thaliana*. Sequences which returned an e-value≤10^−10^ to any sequence of these control proteomes were filtered out. The remaining parasite proteins were run through Phobius and predicted N-terminal export signal sequences were cut off at the predicted cleavage site. Then, the protein sequences were converted to a series of overlapping 14-mers with a sliding window of increment one. The resulting peptides were screened against the five control proteomes with ungapped blastp, and 14-mers above the empirically determined identity threshold (represented by the red line in Figure S2) were removed. With the remaining, parasite-specific 14-mers, an ungapped blastp search was performed against the host proteome and hits above the empirically determined identity threshold (green line in Figure S2) were considered molecular mimicry candidates. Randomized sequences were generated with ‘shuffleseq’ of the EMBOSS package [54]. All programs were run on the University of Bern Linux cluster, Ubelix <http://ubelix.unibe.ch>. Multiple sequence alignments were performed using ClustalX [55].

### Database

The mimicDB database (http://mimicDB.scilifelab.se) uses MySQL as its relational database engine. The database was designed as an extension to the GO term [56] database schema for ease of interrogation on the complete GO hierarchy rather than leaf term only (Figure S3). Protein motif predictions were obtained using hmmer 3.0 [57] with the PFAM database v24.0 [58], and signal peptide predictions using Phobius 1.01 [40]. *Ad hoc* Perl scripts were used to import the mimicry pipeline results, predicted motifs and signals as well as calculate Shannon source entropy for peptides. The interface was constructed using Perl and the Titanium extension to CGI.pm. A package to reconstruct the results and database is available from the authors upon request or can be downloaded from the mimicDB web site.

## Supporting Information

Figure S1 **ClustalW dendrogram of CRIT orthologues from** ***Schistosoma mansoni*** **(Sma),** ***S. haematobium*** **(Sha),** ***S. japonicum*** **(Sja),** ***Trypanosoma cruzi*** **(Tcr),** ***H. sapiens*** **(Hsa), and** ***R. norvegicus*** **(Rno).** The scale bar indicates changes per site. Bootstrapping numbers (grey) are given as percent positives of 1,000 rounds.
(TIF)

Figure S2 **The filtering system used in the overlapping fragments approach.** Numbers represent identical amino acid residues. Red line: threshold for negative control species. Green line: threshold for molecular mimicry candidate in mammalian host or insect vector.
(TIF)

Figure S3 **Database schema of mimicDB.** The mimicDB database schema centers around *mimic_sequence*, which represents the individual genes. This table has as attribute tables the actual peptide sequences (*mimic_sequence_seq*) and predicted motifs (*mimic_sequence_motif*). Hits between parts of these genes are collected in *mimic_hit*, which stores the coordinates and properties of the hit. A complexity measure, in the form of Shannon source entropy for each peptide hit is stored in *mimic_hit_entropy*. The database connects to the GO consortium GO term database in that *mimic_sequence* entries that have a GO association are referenced by entries in *mimic_sequence_with_go_association*, where the corresponding GO term db gene_product::id is also a foreign key.
(TIF)

Table S1 **All molecular mimicry candidates identified searching the human proteome with full-length protein sequences from parasites.** Scores are from blastp searches using the BLOSUM62 matrix and default parameters. Ratios are of the score against *H. sapiens* divided by the best score achieved against any of the control species *Arabidopsis thaliana*, *Caenorhabditis elegans*, *Ciona intestinalis*, *Schizosaccharomyces pombe*, or *Trichoplax adhaerens*.
(XLS)

Table S2 **Molecular mimicry candidates identified searching the human proteome with fragmented protein sequences from parasites.** Hits are sorted according to GO (gene ontology) process annotation of the respective human target protein. Enrichment (‘Enrich’) of GO terms in the identified sets of target proteins is expressed in relation to the abundance of the same GO terms in the complete human proteome (last three columns).
(XLS)
