# Supplementary figures and images for: Genome-Wide Identification of Molecular Mimicry Candidates in Parasites
Source: PLoS One. 2011 Mar 8;6(3):e17546. doi: 10.1371/journal.pone.0017546 (PMC3050887; doi:10.1371/journal.pone.0017546)

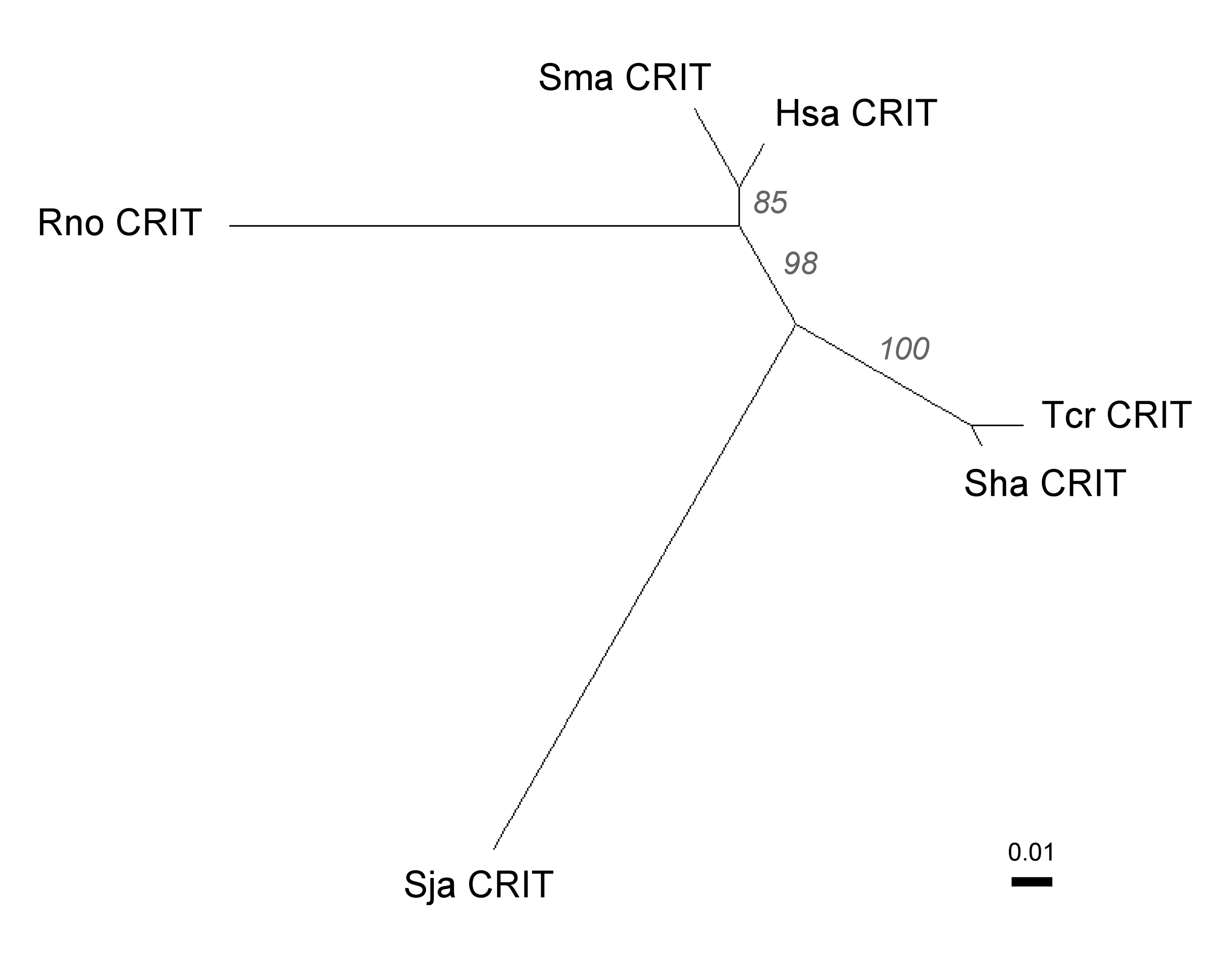

Supplement: Figure S1 — ClustalW dendrogram of CRIT orthologues from Schistosoma mansoni (Sma), S. haematobium (Sha), S. japonicum (Sja), Trypanosoma cruzi (Tcr), H. sapiens (Hsa), and R. norvegicus (Rno). The scale bar indicates changes per site. Bootstrapping numbers (grey) are given as percent positives of 1,000 rounds. (TIF) [file pone.0017546.s001.tif]

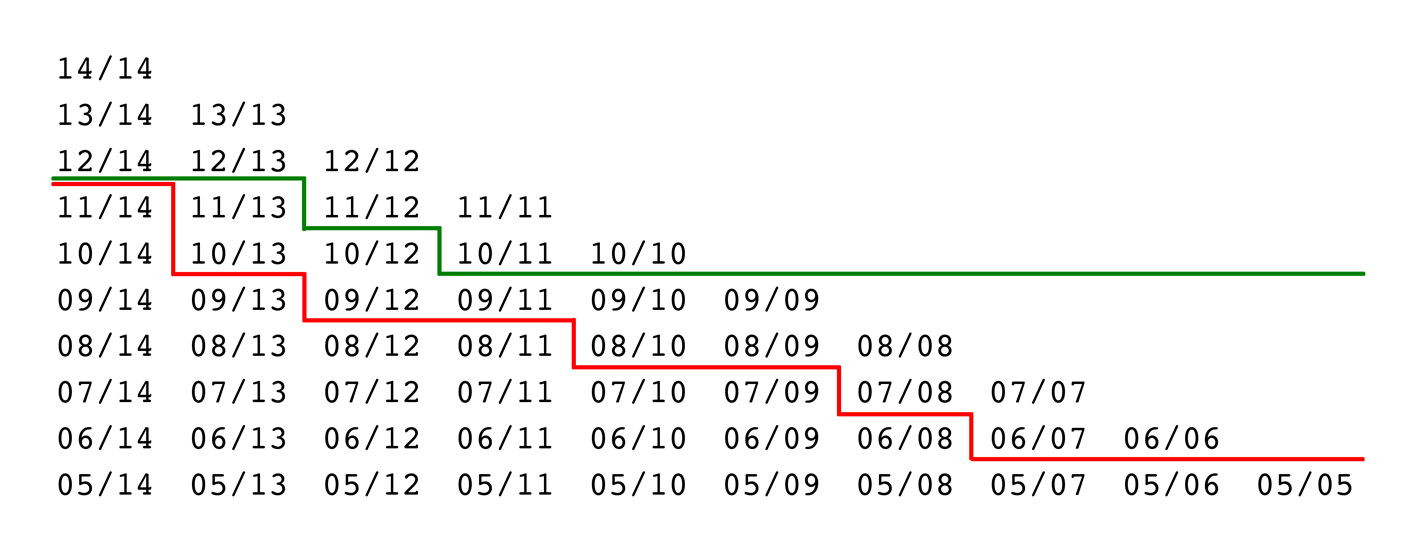

Supplement: Figure S2 — The filtering system used in the overlapping fragments approach. Numbers represent identical amino acid residues. Red line: threshold for negative control species. Green line: threshold for molecular mimicry candidate in mammalian host or insect vector. (TIF) [file pone.0017546.s002.tif]

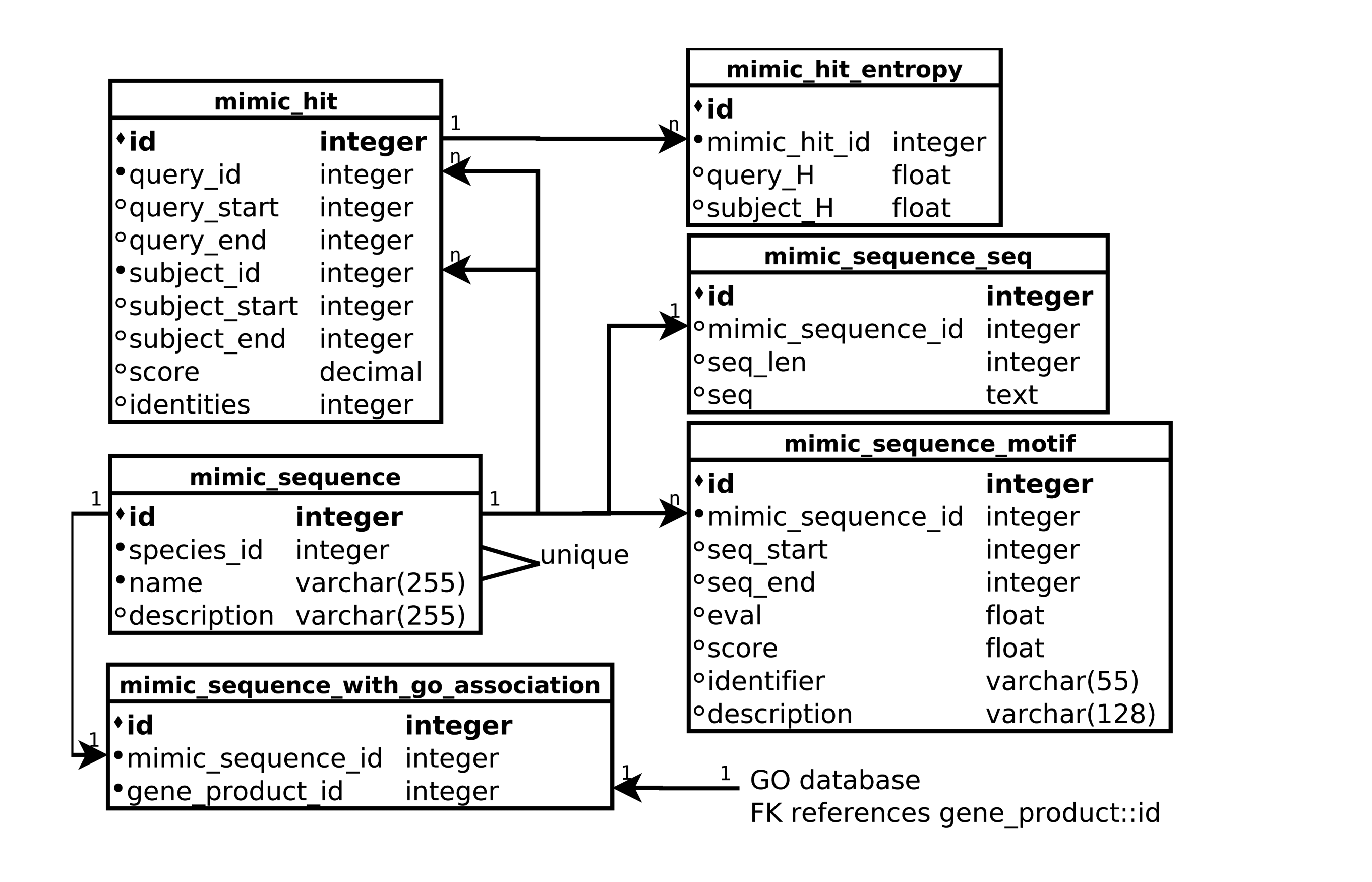

Supplement: Figure S3 — Database schema of mimicDB. The mimicDB database schema centers around mimic_sequence, which represents the individual genes. This table has as attribute tables the actual peptide sequences (mimic_sequence_seq) and predicted motifs (mimic_sequence_motif). Hits between parts of these genes are collected in mimic_hit, which stores the coordinates and properties of the hit. A complexity measure, in the form of Shannon source entropy for each peptide hit is stored in mimic_hit_entropy. The database connects to the GO consortium GO term database in that mimic_sequence entries that have a GO association are referenced by entries in mimic_sequence_with_go_association, where the corresponding GO term db gene_product::id is also a foreign key. (TIF) [file pone.0017546.s003.tif]
